# Emerging Resistance Mechanisms in Gram-Positive Bacteria Isolated From Septicemia Cases in ICUs: A Focus on Genotypic Insights

**DOI:** 10.7759/cureus.76979

**Published:** 2025-01-05

**Authors:** Sharon Christina, Raveendran Praveena, Chitralekha Saikumar

**Affiliations:** 1 Microbiology, Sree Balaji Medical College and Hospital, Bharath Institute of Higher Education and Research, Chennai, IND; 2 Microbiology, Sree Balaji Medical College and Hospital, Bharath Institute of Higher education and Research, Chennai, IND

**Keywords:** bloodstream infections, coagulase-negative staphylococci (cons), methicillin-resistant staphylococcus aureus (mrsa), polymerase chain reaction, septicemia

## Abstract

Background

Bloodstream infections (BSIs) are associated with high morbidity and mortality, especially in intensive care unit (ICU) settings. The most common Gram-positive pathogens, methicillin-resistant *Staphylococcus aureus* (MRSA) and coagulase-negative *Staphylococci *(CoNS), are likely to cause BSIs. The phenotypic and genotypic characteristics of multidrug-resistant (MDR) *Staphylococcal *species isolated from patients admitted to the ICU with septicemia were evaluated for better treatment outcomes.

Materials and methods

A cross-sectional study was conducted at Sree Balaji Medical College and Hospital over two years (July 2022 to June 2024). Blood samples in blood culture bottles received in the laboratory from ICU patients with suspected sepsis were included in this study. The BacT/ALERT® 3D system (bioMérieux, France) was used to assess the bacterial growth. The VITEK^® ^2 system (bioMérieux, France) and conventional methods were used to identify Gram-positive isolates. Antibiotic susceptibility was determined by the Kirby-Bauer disc diffusion method, and polymerase chain reaction (PCR) was used to detect genes like mecA, icaA, and icaD genes.

Results

Out of 274 blood samples, eight (2.9%) were contaminants, and 121 (44.2%) were culture-positive as true pathogens. Eighty-seven (71.9%) Gram-positive isolates were identified from the positive blood cultures, of which CoNS was predominant (51, 58.6%), followed by *Staphylococcus aureus* (25, 28.7%). Methicillin resistance was observed in 10 (13.1%) *Staphylococcus aureus* and 14 (18.4%) CoNS isolates. PCR detected the mecA gene in 20 (83.3%) methicillin-resistant isolates and biofilm-related genes icaD in 64 (84.2%) and icaA in 58 (76.3%). Vancomycin and teicoplanin showed high effectiveness.

Conclusions

This study has emphasized the importance of molecular screening for the mecA, icaA, and icaD genes in framing antibiotic regimens. The findings emphasize the efficacy of vancomycin, teicoplanin, and linezolid in combating MDR *Staphylococcus* infections in ICUs of hospitals and demonstrate the importance of antibiotic stewardship.

## Introduction

Major illnesses known as bloodstream infections (BSIs) have a high mortality and morbidity rate that can be challenging to treat and often cause considerable social and financial hardship [1]. Blood cultures also provide essential information for evaluating a variety of diseases like endocarditis, pneumonia, and pyrexia of unknown origin, particularly in patients with suspected sepsis. Microorganisms in the blood persistently or intermittently pose a significant threat to the host [2]. Gram-positive organisms are the most common infectious agents isolated in BSIs [3]. *Staphylococcus, Streptococcus*, and *Enterococcus* species are the most commonly isolated Gram-positive cocci (GPC) [4].

Antibiotic resistance has been associated with increased rates of clinical burden and mortality burden, prolonged hospitalizations, and high costs associated with healthcare, leading to an increasing worldwide health concern [5]. If antibiotic resistance remains unresolved, it is estimated that 300 million people will perish due to resistance to antibiotics over the next 35 years [2]. Increasing multidrug resistance to Gram-positive bacteria, especially *Staphylococci, Streptococci, *and *Enterococci, *is an alarming problem as it limits treatment options and makes infection control difficult [6]. The availability of newly developed antibiotics that are effective against Gram-positive bacteria has remained extremely limited in recent years. Bacterial strains with non-susceptibility to one or more antimicrobials in three or more antimicrobial classes are defined as multidrug-resistant (MDR) [4]. Common bacterial infections have become more difficult and sometimes impossible to manage with common antibiotics because of the significant rise in MDR strains over the past few years [7].

Globally, *Staphylococcus aureus* is one of the most prevalent Gram-positive MDR bacteria responsible for nosocomial infections [6]. It frequently results in infections in healthy individuals and those with associated risks or underlying health conditions, both in healthcare facilities and the community [8]. From superficial skin infections to serious medical conditions like pneumonia, osteomyelitis, sepsis, and bacteremia, *Staphylococcus aureus *can cause an extensive range of infections [9]. The Centers for Disease Control and Prevention and the Public Health Agency of Canada consider methicillin-resistant *Staphylococcus aureus* (MRSA) to be a major risk for serious conditions like skin and soft tissue infection, pneumonia, endocarditis, sepsis, and septic shock [8]. Based on previous studies, individuals with MRSA infections have a 1.19-fold increase in hospital expenditure and a greater 30/90-day risk of mortality than patients with MSSA infection [10]. This study examined the phenotypic and genotypic characteristics of MDR Gram-positive cocci isolated from blood culture samples of patients admitted to the intensive care unit (ICU) with septicemia in a tertiary care hospital.

## Materials and methods

Isolation of bacteria

This is a cross-sectional study that was conducted over a period of two years, from July 2022 to June 2024. The blood samples were obtained from patients admitted to the ICU with a diagnosis of septicemia at Sree Balaji Medical College and Hospital, Chennai.

Blood samples were collected in a blood culture bottle containing brain heart infusion broth for bacterial isolation and were received in the Department of Microbiology, Central Laboratory. The samples were incubated at 37℃ for five to seven days using the BacT/ALERT® 3D system (bioMeriéux, France). The positive blood culture bottles were removed from the BacT/ALERT® 3D system as soon as they were flagged as positive by the system, and a direct smear was prepared from the culture bottle. Gram stain was performed from the smear and focused under a microscope, and all the GPC were subcultured on blood agar, nutrient agar, and mannitol salt agar and incubated at 37°C for 24 to 48 hours. Microbial identification was performed with standard conventional methods like the Kirby-Bauer disc diffusion method in conjunction with the VITEK® 2 system (bioMérieux, France).

Inclusion and exclusion criteria

Patients from all age groups diagnosed with septicemia were included in this study. Patients with a history of antimicrobial therapy in the last one week and samples with evidence of contamination were excluded from this study.

True pathogens were identified when (1) the same species was isolated from two or more sets of blood cultures, (2) MDR resistance was observed with clinical significance, and (3) virulence genes and biofilm formation were detected.

Antibiotic sensitivity test

The antibiotic susceptibility test was performed using Kirby-Bauer's method, which was in line with the guidelines provided by the Clinical and Laboratory Standards Institute (CLSI). With the lawn culture method, an inoculum was prepared for each bacterial isolate by adjusting its turbidity to the 0.5 McFarland standard and was inoculated on the Mueller-Hinton agar plate. Antibiotic-containing discs (Himedia Laboratory) were placed on the surface of the lawn culture and incubated for 24 hours at 37°C. The zone of inhibition has been classified as sensitive, intermediate, or resistant to the tested antibiotics according to the CLSI M100 Guideline 2022.

Polymerase chain reaction

Polymerase chain reaction (PCR) amplification was done for all phenotypically detected MRSA isolates to detect the genes encoding resistance mecA [11], icaA [12], and icaD genes. Table 1 shows PCR primer sequences for mecA, icaA, and icaD genes, producing amplicons of 255 bp, 188 bp, and 198 bp, respectively.

**Table 1:** Primer details F: forward, R: reverse

Gene	Sequence	Product size
mecA [11]	F 5–AAAATCGATGGTAAAGGTTGGC-3 R 5-AGTTCTGGAGTACCGGATTTGC- 3	255 base pairs
icaA [12]	F 5-ACACTTGCTGGCGCAGTCAA-3 R 5-TCTGGAACCAACATCCAACA-3	188 base pairs
icaD	5-ATGGTCAAGCCCAGACAGAG-3 5-AGTATTTTCAATGTTTAAAGCAA-3	198 base pairs

Statistical analysis

The statistical analysis was performed using Microsoft Excel 2023 (Microsoft Corporation, Redmond, USA) to enable effective sorting and filtering; the data, which included patient demographics, bacterial species, resistance to antibiotics, and genotypic markers, was put together in structured tables. Descriptive statistics like averages and percentages were computed to compile resistance patterns among isolates.

Ethical consideration

The Institutional Human Ethics Committee of Sree Balaji Medical College and Hospital, Bharath Institute of Higher Education and Research, Chennai, issued approval number 002/SBMCH/IHEC/2022/1720.

## Results

In this study, a total of 274 cases with septicemia who were admitted to the ICU were included, and isolates from the blood samples of those patients were taken for further study. Of 274 blood samples, 178 (64.9%) were from male patients, and 102 (37.2%) were from female patients. A maximum number of samples were received from the age group of 40-60 (90, 32.8%), and the minimum samples were obtained from the less than 10 years of age group (18, 6.5%). Patients had various comorbidities; especially 68 (24.8%) of the patients had type 2 diabetes mellitus, which could be a risk factor for septicemia, followed by systemic hypertension (55, 20.0%). The types of treatment received by the patient during a hospital stay are invasive procedures (77, 28.1%), prior antibiotics (123, 44.8%), and implants (7, 2.5%), which can significantly contribute to the development of MDR bacteria.

Out of 274 blood samples, eight (2.9%) were contaminants, and 121 (44.2%) were culture-positive as true pathogens. We isolated 87 (71.9%) GPC isolates from the culture-positive samples, and the remaining were Gram-negative bacilli and *Candida* species. The predominant species of identified GPC was coagulase-negative *Staphylococci *(CoNS)* *species (51, 58.6%), followed by *Staphylococcus aureus* (25, 28.7%), *Streptococcus *species (6, 6.8%), and *Enterococcus *species (5, 5.7%).

**Figure 1:**
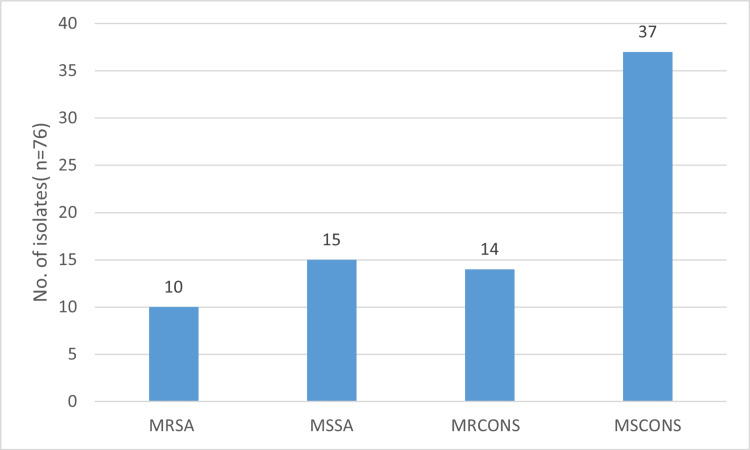
Methicillin sensitivity pattern of Staphylococcus species MRSA: methicillin-resistant *Staphylococcus aureus, *MSSA: methicillin-susceptible *Staphylococcus aureus, *MRCoNS: methicillin-resistant coagulase-negative *Staphylococci, *MSCoNS: methicillin-sensitive coagulase-negative* Staphylococci*

Among 51 CoNS* *species, *Staphylococcus epidermidis* (23, 45%) was found to be more prevalent, followed by *Staphylococcus hemolytic* (12, 23.5%), *Staphylococcus hominis* (9, 17.6%), *Staphylococcus saprophyticus* (4, 7.8%), and *Staphylococcus capitis* (3, 5.8%). We observed that out of 76 GPC isolates, 10 (13.2%) were MRSA, and 14 (18.4%) were methicillin-resistant CoNS (MRCoNS), as shown in Figure 1.

Table 2 shows that out of 24 methicillin-resistant *Staphylococcus *species, the mecA gene was found in 9 (37.5%) MRSA and 11 (45.8%) MRCoNS isolates.

**Table 2:** Genotypic characterization of MRSA and MRCoNS among the isolates MRSA: methicillin-resistant *Staphylococcus aureus, *MRCoNS: methicillin-resistant coagulase-negative *Staphylococci*

Drug-resistant genes	MRSA no. of isolates (%)	MRCoNS no. of isolates (%)	Total no. of isolates (%)
MecA positive	9 (37.5)	11 (45.8)	20 (83.3)
MecA negative	1 (4.1)	3 (12.5)	4 (16.7)
Total	10 (41.6)	14 (58.3)	24

Among 76 *Staphylococcus* species, 64 (84.2%) yielded the icaD gene; 58 (76.3%) strains were presented with the icaA gene, as shown in Table 3.

**Table 3:** Gene distribution of icaD and icaA genes among the isolates

No. of bacterial isolates (%)	No. of isolates with icaD gene (%)	No. of isolates with icaA gene (%)
76	64 (84.2)	58 (76.3)

**Figure 2:**
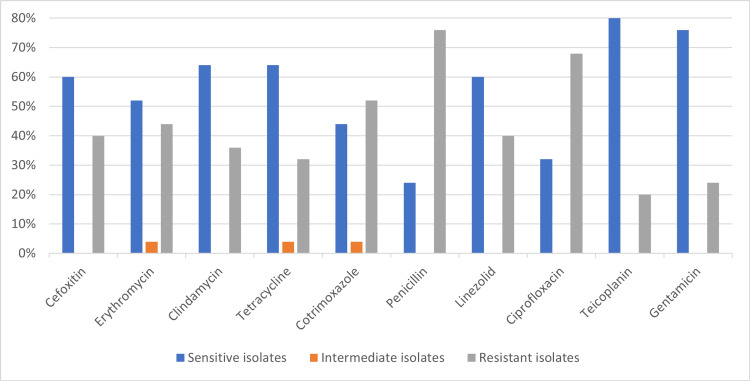
Antibiotic susceptibility pattern of Staphylococcus aureus

*Staphylococcus aureus *susceptibility pattern showed sensitivity of cefoxitin (15, 60%), erythromycin (13, 52%), clindamycin (16, 64%), tetracycline (16, 64%), cotrimoxazole (11, 44%), penicillin (6, 24%), linezolid (15, 60%), ciprofloxacin (8, 32%), teicoplanin (20, 80%), and gentamicin (19, 76%), as shown in Figure 2.

**Figure 3:**
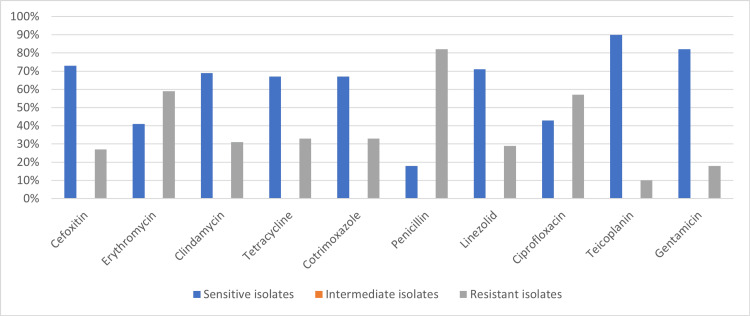
Susceptibility pattern of CoNS (MSCoNS and MRCoNS) MRCoNS: methicillin-resistant coagulase-negative *Staphylococci*; MSCoNS: methicillin-sensitive coagulase-negative *Staphylococci*

The susceptibility of CoNS showed a sensitivity of cefoxitin (37, 73%), erythromycin (21, 41%), clindamycin (35, 69%), tetracycline (34, 67%), cotrimoxazole (34, 67%), penicillin (9, 18%), linezolid (36, 71%), ciprofloxacin (22, 43%), teicoplanin (46, 90%), and gentamicin (42, 82%), as seen in Figure 3.

**Figure 4:**
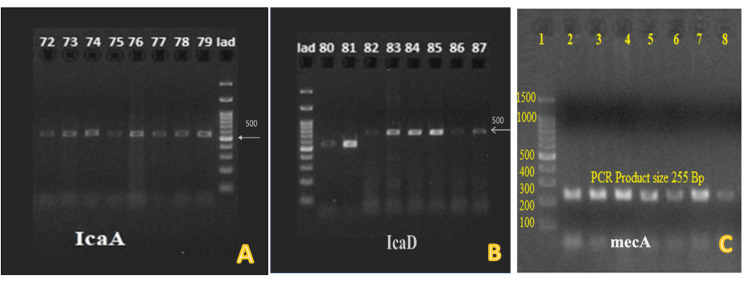
Gel electrophoresis profile for the detection of MRSA The icaA, icaD, and mecA genes detected by PCR are responsible for MDR PCR: polymerase chain reaction, MDR: multidrug-resistant, MRSA: methicillin-resistant Staphylococcus aureus

In Figure 4, image A shows isolates 72-74 and 76-79 positive for the icaA gene with a 500 bp bandwidth in the DNA ladder. Image B shows isolates 81, 83, 84, 85, and 87 positive for the icaD gene with a bandwidth of 500. Image C shows two to seven positive isolates for the mecA gene with 255 bp in the DNA ladder.

**Figure 5:**
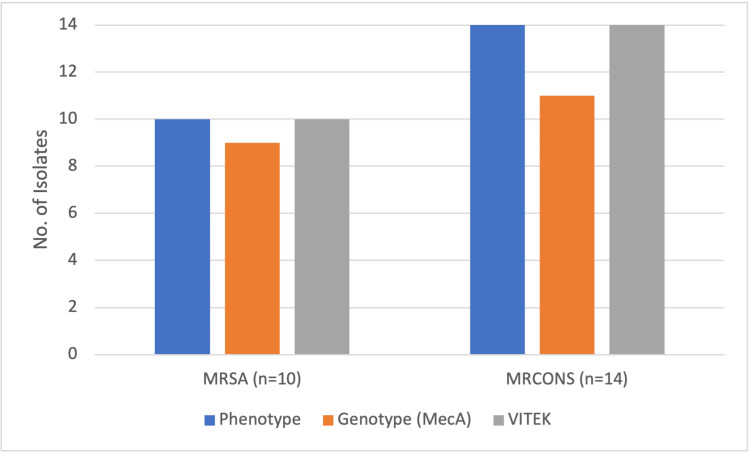
Phenotypic and genotypic characterization of methicillin-resistant Staphylococcal strains MRSA: methicillin-resistant *Staphylococcus aureus, *MRCoNS: methicillin-resistant coagulase-negative *Staphylococci*

The phenotypic and automated VITEK® 2 system identified all 24 methicillin-resistant *Staphylococcus* species, but the genotypic method using the mecA gene could identify only 20 (83.3%) strains out of 24 isolates, as shown in Figure 5.

## Discussion

Gram-positive bacteria exhibit two main ways to develop resistance: either by producing β-lactamases, which degrades β-lactam antibiotics, or by decreasing their affinity and susceptibility to the antibiotic's target site, the penicillin-binding protein (PBP), through the acquisition of exogenous DNA or through modifying the PBP genes [13]. *Staphylococcus aureus *strains that are resistant to methicillin and the misuse of other antibiotics lead to a vast majority of infections in healthcare units worldwide. As it encodes the low-affinity PBP 2A, the mecA gene causes methicillin resistance [14]. Infections associated with catheters and other medical devices are frequently caused by *Staphylococcus aureus* and *Staphylococcus epidermidis*. Recent studies have demonstrated that *Staphylococcus aureus*, which carries the ica operon that regulates slime synthesis, can produce slime similar to *Staphylococcus epidermidis*. Co-expression of the icaA and icaD genes is required for complete slime synthesis in the operon [15].

In our study, we observed among 87 GPC isolates, the predominant species was coagulase-negative *Staphylococcus *species (51, 58.6%), followed by *Staphylococcus aureus* (25, 28.7%). Out of 76 *Staphylococcus* species, the most common organisms were *Staphylococcus aureus *(25, 32.8%), *Staphylococcus epidermidis* (23, 30.2%), *Staphylococcus hemolyticus* (12, 15.7%), *Staphylococcus hominis* (9, 11.8%), *Staphylococcus saprophyticus* (4, 5.2%), and *Staphylococcus capitis* (3, 3.9%).

Distribution of susceptibility and drug-resistant Gram-positive cocci

*Staphylococcus aureus* showed high resistance rates to penicillin (18, 72%), followed by ciprofloxacin (16, 64%) and cotrimoxazole (13, 52%). Likewise, CoNS showed high resistance to penicillin (42, 82.3%), ciprofloxacin (29, 56.8%), and erythromycin (30, 58.8%). Our study showed that 24 (31.5%) *Staphylococcus* species were identified as methicillin-resistant through the phenotypic method and automated VITEK® 2 system. Of 24 methicillin-resistant *Staphylococcus *species, 10 (41.6%) were identified as MRSA, and 14 (58.3%) were MRCoNS.

Distribution of polymerase chain reaction mecA, icaA, and icaD genomes

In this study, we observed that among 24 methicillin-resistant Staphylococcus species, the mecA gene was found in nine (37.5%) MRSA and 11 (45.8%) MRCoNS isolates. It is also reported that among 76 *Staphylococcus* species, 64 (84.2%) of them yielded the icaD gene. Meanwhile, 58 (76.3%) strains were presented with the icaA gene, as shown in Table 2.

In a study by Rocchetti et al., CoNS was identified in 286 (77.0%) blood cultures and *Staphylococcus aureus* in 85 (23.1%). Among 85 *Staphylococcus aureus*, 43 (50.6%) had the mecA gene, whereas 225 (78.7%) of the 286 CoNS showed positive for the mecA gene, highlighting the strong association of the mecA gene responsible for methicillin resistance [16]. Our study showed a high prevalence in the icaD and icaA genes responsible for biofilm production. In our study, the icaD and icaA genes were 64 (84.2%) and 58 (76.3%), respectively. This indicates that *Staphylococcus* species have an increased tendency to produce biofilms.

Another study performed by Idrees et al. showed that among 63 MRSA isolates, 56 (88.8%) were found to have the mecA gene, whereas seven (11.1%) had no evidence of the mecA gene. In the antimicrobial sensitivity test, these bacterial isolates exhibited absolute resistance to cefoxitin and penicillin [17], similar to our study. In a study by Satorres and Alcaráz, PCR detected icaA and icaD genes in 19 (35.2%) clinical strains of *Staphylococcus aureus* [18]. However, Fowler et al. found that every *Staphylococcus aureus* isolate had icaA and icaD positive [19]. Based on another study by Arciola et al., *Staphylococcus aureus *strains formed biofilm, and the icaA and icaD genes were positive. Each saprophytic strain has been identified to be non-slime-producing and negative for both the icaA and icaD genes [20].

In a study by Nasr et al., 34 (68%) *Staphylococcal* isolates did not have the icaAD gene, whereas 16 (32%) were detected to have the icaD gene [21]. Studies have shown that the formation of biofilm in *Staphylococc*i causing catheter-associated and nosocomial infections is associated with the presence of both icaA and icaD genes in different proportions [22]. Our study proves this hypothesis by demonstrating that biofilm formation increases the resistance and persistence of infections associated with medical devices and depends on the co-expression of icaA and icaD genes. Even though phenotypic biofilm formation has not been studied, the significant genotypic prevalence of icaA and icaD in our isolates suggests that these genes are crucial in understanding the antimicrobial resistance pattern of infectious diseases associated with biofilms.

Finally, we postulate that studying the presence of adhesion molecule genes, like the icaA and icaD genes, may help identify the role of different adhesion processes in the course of infection associated with medical devices. When the icaA and icaD genes function together, biofilm formation is facilitated, thereby increasing their ability to adhere to the host surfaces and enhancing bacterial antibiotic resistance. 

Identifying the mecA, icaA, and icaD genes is essential for enhancing clinical therapy approaches and developing hospital antibiotic policies. Methicillin resistance, caused by the mecA gene, emphasizes the need for alternative antibiotics, such as vancomycin or linezolid, to treat resistant strains and decrease beta-lactam misuse. Genes such as icaA and icaD, which constitute the formation of biofilms, indicate the possibility of chronic infections, allowing clinicians to utilize combination or dose-optimized treatment to eliminate biofilms [23]. This screening for genes helps prevent the development of antibiotic-resistant infections in healthcare facilities, encourages timely interventions, allows precise, effective treatments, and enhances infection control.

Limitations

Two hundred seventy-four blood samples from a single tertiary care hospital have been included in the study, suggesting they might not accurately represent the general population. The understanding of biofilm-related resistance mechanisms has been limited since phenotypic confirmation of biofilm formation was not carried out despite collecting genotypic data on biofilm-related genes (icaA, icaD).

## Conclusions

The present study showed the prevalence of bacterial pathogens in BSIs and their antibiotic sensitivity patterns. Specific antibiotic utilization strategies, such as antibiotic restriction, combination therapy, antibiotic usage according to standard antimicrobial susceptibility testing, and antibiotic recycling, may help reduce the incidence of BSIs and prevent the emergence of drug resistance.

In light of the reduced resistance rates, vancomycin, linezolid, teicoplanin, and clindamycin can be utilized, according to the current study, to treat MRSA infections. Clinical settings should routinely investigate the mecA gene in MRSA molecularly. In addition to impacting novel MRSA treatment approaches, the evolving molecular basis of MRSA drug resistance makes it more difficult to prevent MRSA infections. Further epidemiological and molecular studies are necessary to better understand the emergence of multidrug resistance in MRSA and prevent the spread of *Staphylococcus aureus* in nearby regions. Furthermore, the presence of the icaA and icaD genes in *Staphylococcus *species is gradually increasing, suggesting that a more accurate method of biofilm detection may be provided through genotypic screening for the above genes and phenotypic techniques. Regularly assessing the icaA and icaD genes could also help successfully execute focused control measures for infections associated with biofilms.
